# Preaxial Polydactyly in an Elderly Woman

**DOI:** 10.1155/2022/7031401

**Published:** 2022-08-31

**Authors:** Barkha Chhabra, Richy Charls, John J. Faillace

**Affiliations:** ^1^Department of Orthopaedic Surgery and Rehabilitation, The University of Texas Medical Branch, 301 University Blvd Route 0165, Galveston, TX 77555-0165, USA; ^2^School of Medicine, The University of Texas Medical Branch, 301 University Blvd, Galveston, TX 77555, USA

## Abstract

A 70-year-old woman born and raised in India presented with Wassel type IV preaxial polydactyly of the right thumb and difficulty performing daily activities. She elected for surgical reconstruction after postponing the procedure for many years due to cultural norms. Her postoperative course was unremarkable. At her 3-month follow-up, she was diagnosed with carpal tunnel syndrome and underwent open carpal tunnel release. Preaxial polydactyly repair is unusual in patients older than 25 years. Though the duplication is considered lucky in South Asia, indications for this case were arthritic pain, cosmesis, and function. This case report details a unique indication for polydactyly reconstruction, arthritic pain, which may benefit hand surgeons when discussing the literature on adult polydactyly with their patients.

## 1. Introduction

Preaxial polydactyly refers to various developmental errors along the anteroposterior axis of the upper limb [1]. Incidence of this condition ranges from 1 in 1000 to 1 in 10,000 but can differ greatly from various geographic regions and is more common in males. This defect in normal embryonic development leads to numerous duplicated anatomical possibilities, ranging from bifid distal phalanxes to triphalangeal first digits. Wassel's classification system of thumb polydactyly is a radiographic description based on the level of duplication, where a Roman numeral is assigned based on how proximally the bifurcation or duplication extends, with greater numbers describing more proximal levels of duplication. Odd numbers represent bifid osseous structures, and even numbers indicate duplicated bones [2]. Wassel's manuscript provided recommendations regarding operative technique and timing of surgery for each type [2]. Many reconstruction methods have been described, all of which include surgical ablation of the supernumerary digit followed by reconstruction of ligaments for stability to restore function and acceptable cosmesis [2]. Historically, reconstruction was performed between the ages of 1 and 2 years old, although some current recommendations are to delay the reconstruction until after age 2 due to possible effects of anesthesia on the developing brain [3]. We present a case of preaxial polydactyly in a 70-year-old woman, representing a peculiar demographic for this type of operation.

## 2. Case Presentation

**Figure 1:**
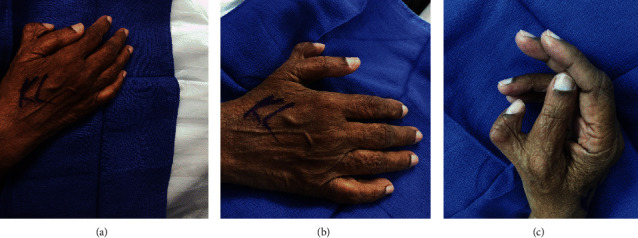
Preoperative clinical photographs demonstrating preaxial polydactyly of the patient's right thumb. (a) Oblique view. (b) Dorsal view. (c) Lateral view.

Written informed consent was obtained from the patient for publication of this case report and accompanying images. A 70-year-old, right hand-dominant woman presented to our general orthopaedic clinic initially for evaluation of lumbar back pain. The team noted the presence of an extra digit involving her right hand, specifically preaxial polydactyly of her right thumb (Figures 1(a)–1(c)). The patient reported increasing sensitivity to cold temperatures in the extra digit, as well as impairment of routine daily activities, such as utilizing gloves and ambulating with her walker, due to painful pressure on the digit. Because of this impairment of function, she was referred for consultation to the hand team.

**Figure 2:**
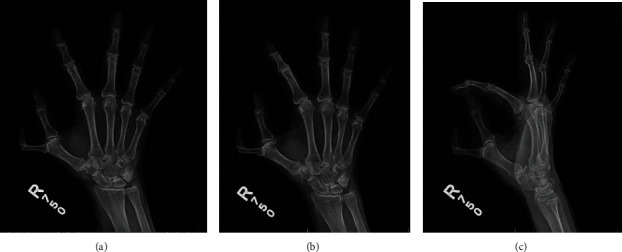
A 3-view X ray of the right hand showed preaxial polydactyly. (a) Duplicated proximal and distal phalanx, anterior-posterior view. (b) Subchondral sclerosis, oblique view. (c) Reactive bone formation suggestive of arthritic changes at the radial preaxial polydactyly MCP joint (Wassel type IV), lateral view.

Physical exam of the hand revealed limited range of motion of the radial thumb, which was fixed in 90 degrees of flexion. The ulnar thumb, which extended to 0° and flexed to 50° at the interphalangeal (IP) and metacarpophalangeal (MCP) joints, appeared to be dominant and more functional. Sensation was intact in median, ulnar, and radial distributions, including the medial and lateral borders of the extra digit, and motor function was normal with full extension of the wrist and MCP/IP joints as well as full flexion of the wrist and MCP/IP joints of the remaining fingers (2 through 5). A 3-view X ray of the right hand showed preaxial polydactyly with a duplicated proximal phalanx, as well as subchondral sclerosis and reactive bone formation suggestive of arthritic changes at the radial preaxial polydactyly MCP joint due to being apex radial (Wassel type IV) (Figures 2(a)–2(c)). Operative and nonoperative measures were discussed with the patient. Because of her functional impairment and pain, both the patient and the hand surgery team agreed upon surgery as the best course of action. Moreover, the patient remarked that while in India the duplication was considered a sign of good fortune and a blessing, she had become increasingly self-conscious of the deformity after she immigrated to the United States as an adult due to prolonged stares and questions.

**Figure 3:**
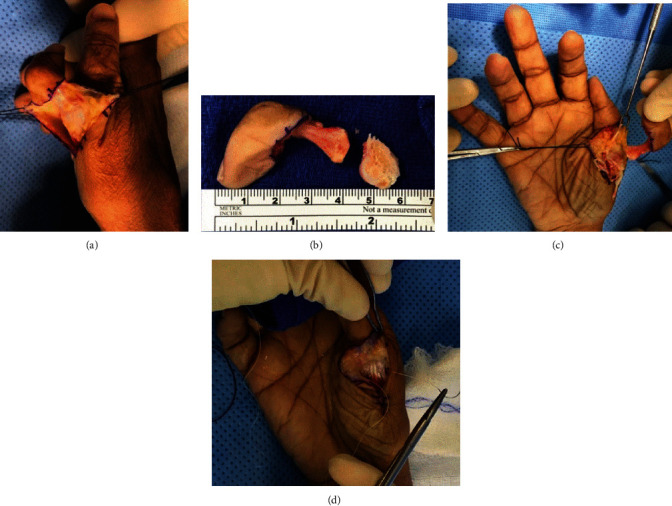
Intraoperative photo of procedure. (a) Thenar racquet incision made with a 15 blade around the extra digit and down the radial side at the junction between the palmar and dorsal skin. (b) Disarticulated extra digit, measuring approximately 6 cm, excised after the extensor and flexor tendons were sharply released distally. (c) Radial facet of the radial thumb joint needing to be removed. Residual capsule was elevated off of the metacarpal base (origin of RCL), and a saw was then used to remove the articular surface and dorsal osteophyte. (d) Approximation of the tendinous insertion of the thenar musculature onto the dorsoradial capsule of the residual proximal phalanx using 3-0 FiberWire®.

Under tourniquet control for hemostasis, a racquet incision was made around the extra digit and down the radial side, at the junction between the palmar and dorsal skin (Figure 3(a)). Dissection was taken down to the insertion of the thenar musculature, and residual extensor and flexor tendons were identified. The periosteum and joint capsule were incised, and the thenar musculature was elevated off the proximal phalanx. The extensor and flexor tendons were sharply released distally, and the extra digit was disarticulated (Figure 3(b)). Residual capsule was elevated off of the metacarpal base, and a saw and rasp were then used to remove the articular surface and dorsal osteophyte (Figure 3(c)).

Using 3-0 FiberWire® (Arthrex, Naples, Florida, USA), a Krakow stitch was placed into the remaining proximal phalanx joint capsule. The wire was threaded through a 2.5 mm PushLock® anchor (Arthrex, Naples, Florida, USA) and the joint capsule tensioned appropriately. The anchor was then placed at the isometric point on the radial aspect of the metacarpal head. A tendon passer was used to pass the residual extensor tendon up through the capsule and onto the volar aspect of the capsule. The nerve to the amputated digit was implanted in the thenar musculature. The tendinous insertion of the thenar musculature was then approximated onto the dorsoradial capsule of the residual proximal phalanx using 3-0 FiberWire (Figure 3(d)). The incision was closed in layers. After tourniquet release, the incision was appropriately dressed and splinted. The patient was in the OR for 87 minutes.

**Figure 4:**
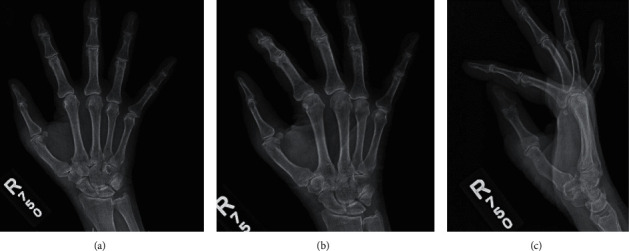
A 3-view X ray of the right hand taken one month postoperatively. (a) Interval resection of the extra digit, dorsal view. (b) Mild degenerative osteoarthrosis of the MCP, oblique view. (c) Mild degenerative osteoarthrosis of the IP joints, lateral view.

Postoperatively, the patient was placed in a thumb spica splint and was advised for no pinching, gripping, or lifting with the right upper extremity to prevent excess ulnar deviation of the thumb. Her postoperative course was uncomplicated, and she was discharged later the same day with return to clinic scheduled for 2 weeks after the procedure. At her first follow-up visit, she reported a small amount of pain and swelling at the incision, as well as phantom sensations of her removed thumb. Physical exam revealed preserved sensory function of the hand and a stable radial collateral ligament at the thumb MCP joint, but decreased range of motion of the reconstructed first digit, with 0° extension and 30° flexion at the MCP joint. Sutures were removed and Steri-Strips applied over the clean, dry, and intact wound. The patient was referred for occupational therapy to improve range of motion. Heat therapy as needed and ibuprofen 800 mg to be taken up to 3 times daily were recommended for postoperative pain control. A 3-view X ray of the right hand demonstrated interval resection of the extra digit with stable, mild, degenerative osteoarthrosis of the first MCP (Figures 4(a)–4(c)). At her 3-month follow-up, she was found to have developed carpal tunnel syndrome and underwent open carpal tunnel release.

**Figure 5:**
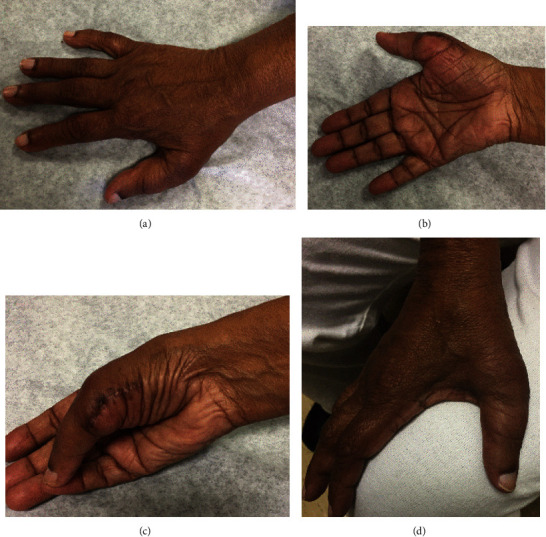
Seven-week postoperative clinical photos demonstrating an attractive cosmetic result. (a) No visible scar dorsally. (b) Minimal visible scar over the volar radial aspect of the right thumb. (c) Full opposition of the right thumb to the distal fifth phalanx. (d) Twelve-week postoperative visit with minimal scar formation and satisfactory thumb abduction.

Her postoperative course was unremarkable, with restoration of good function and significant pain relief, and she was satisfied with the cosmetic improvement of the procedure (Figures 5(a)–5(d)), pain relief, and improved ability to carry out functions of daily living at one year.

## 3. Discussion

Polydactyly can manifest in 3 distinct forms based on where embryological defects occur in relation to the anteroposterior axis of development: preaxial, postaxial, or central axial, with postaxial as the most common and central as the least common. Numerous genes have been implicated in preaxial polydactyly; in particular, errors in the regulatory region (the ZRS) of SHH have been shown to cause preaxial polydactyly. GLI3 and SALL1 have also been shown to cause preaxial polydactyly [4]. Although the frequency can vary greatly among populations, with an incidence ranging from 1 per 1,000 to 10,000 live births, preaxial polydactyly most commonly presents as a Wassel type IV duplication of the proximal phalanx. The usual treatment protocol for these lesions involves early removal; all lesions previously described in the literature were removed before the age of 30 due to functional limitation or gross cosmetic deformity. Gholson et al. [5] conducted a cohort study involving a 35-year clinical and radiographic follow-up of patients undergoing reconstruction procedures for preaxial polydactyly. It was found that patients tended to have significant range-of-motion deficits and mild to moderate functional limitations, as evidenced by the DASH score of 29.5—a full standard deviation below the expected population mean presurgery [5].

A unique aspect of this case includes the ability of our patient to have no functional complaints with the extra radial digit for the greater part of her life. Compared to most other patients undergoing reduction, our patient did not have significant functional limitations until much later in life. The primary reason she sought surgery was for pain relief from arthritic changes, as well as the initial stages of functional loss, such as difficulty ambulating with her walker. This is in stark contrast to the indications for the procedure at earlier ages: early functional impairment, severe pain, or gross cosmetic deformity. de Almeida [6] determined that the best timeframe for surgical correction without the risk of residual deformity was before age 3. Indeed, several sources have stated that reconstruction for polydactyly should not be delayed past age 5 [7]. However, our patient emerged postoperatively with excellent functional and aesthetic outcomes.

The heritage of the patient, who immigrated to the United States from India, as well as cultural values on what constitutes a “deformity” should also be considered. Preaxial polydactyly has an incidence of 1 in 3000 in India, one of the highest in the world [8]. Numerous longitudinal studies have shown that this phenomenon runs in families, with one particular study revealing consistent hand and foot abnormalities throughout 5 generations [9]. In our patient's history, her brother lacked a thumb at birth, while she bore an extra digit. She remarked to us that in her native culture in India, the presence of an extra digit is seen as a harbinger of good fortune. While this belief is not held by the patient, other myths and superstitions in India oppose the removal of an extra digit over fears of divine retribution, as detailed by Dwivedi [10]. This is an interesting contrast to the cultural appraisal of polydactyly in the Western world, in which surgery for this condition is not often delayed after initial diagnosis because of negative psychosocial consequences and isolating effects on children, which can be detrimental to function and overall well-being [11].

Preaxial polydactyly is a condition often treated early in life because of obvious undesirable cosmetic appearance or functional impairment. Our case of preaxial polydactyly in this elderly patient is a unique contribution to the literature and raises intriguing questions of what determines beauty and cosmetic acceptance between different cultural norms.

## Data Availability

No data were used to support this study.
